# Supplementary figures and images for: Transkingdom network reveals bacterial players associated with cervical cancer gene expression program
Source: PeerJ. 2018 Sep 19;6:e5590. doi: 10.7717/peerj.5590 (PMC6170155; doi:10.7717/peerj.5590)

Shannon Diversity Index

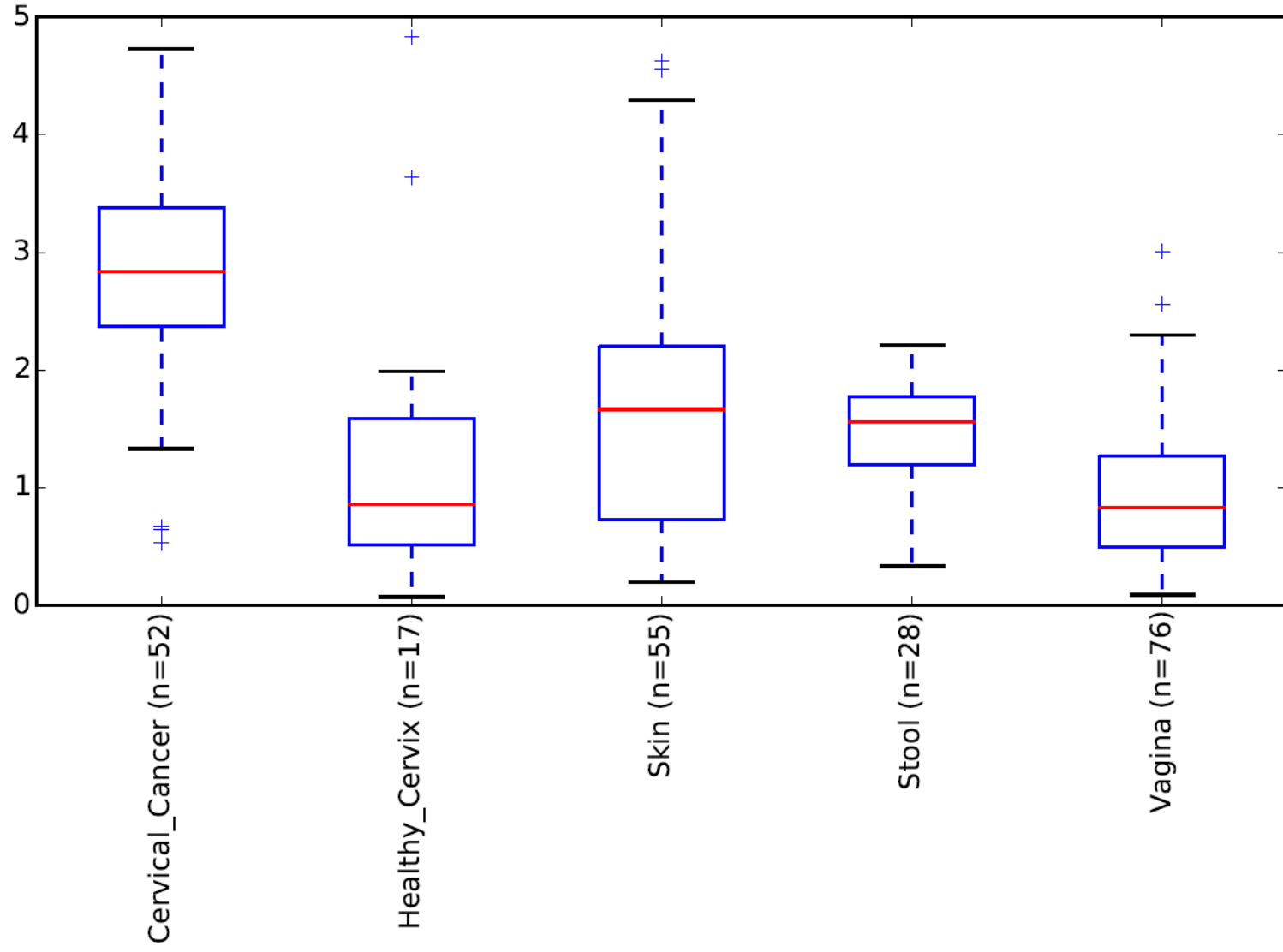

Supplement: Supplemental Information 1 [file peerj-06-5590-s001.pdf]

**A**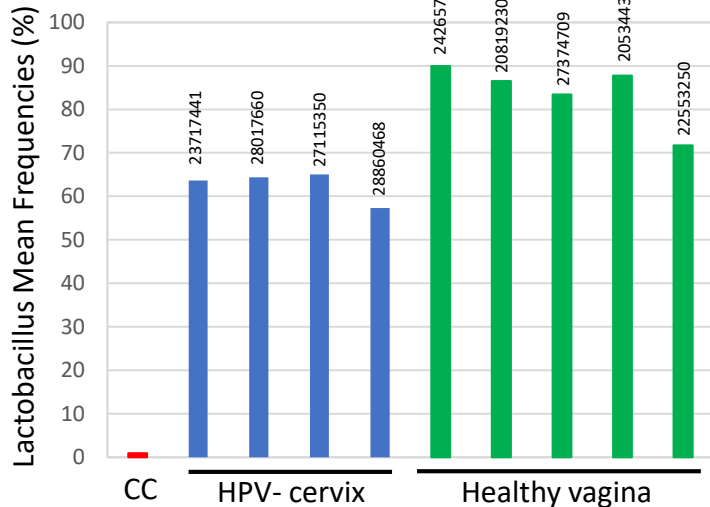**B**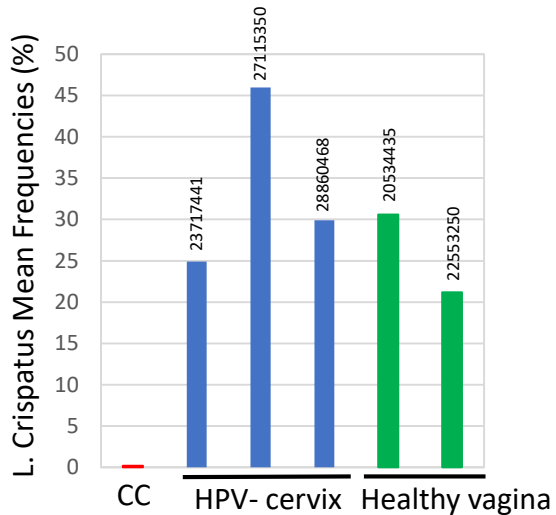

Supplement: Supplemental Information 2 — Abundances of Lactobacillus (A) and L. crispatus (B) in cervical cancer (CC) (our data), HPV negative cervix (HPV-cervix) and healthy vaginal microbiome (Healthy vagina). Abundances are represented by mean frequencies (%) data reported by published articles (PMID numbers of the source article specified for each column). [file peerj-06-5590-s002.pdf]

BTN3A2

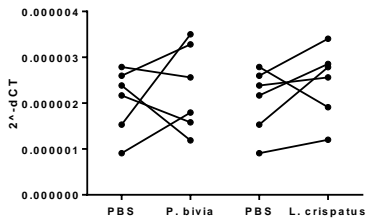

HLA-F

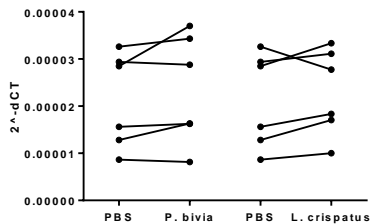

IFI44L

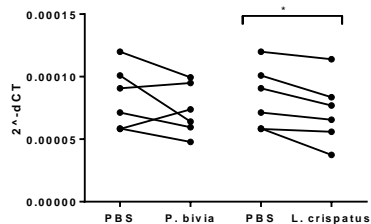

IFI14

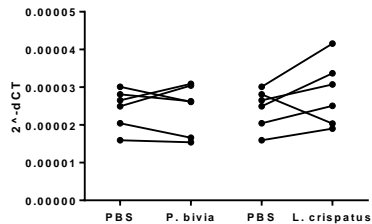

DDN

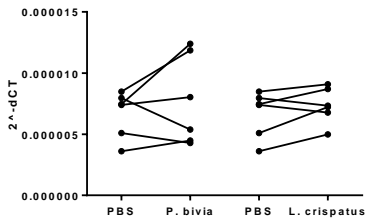

Supplement: Supplemental Information 3 — HeLa cells were co-cultured with either P. bivia or L. crispatus and gene expression was compared to negative treatment (PBS) of Hela cells. mRNA levels were normalized to 18S rRNA gene expression. (*p-value < 0.05, one-tailed Wilcoxon matched-pairs signed rank test). [file peerj-06-5590-s003.pdf]
